# Comparative Outcomes of Beta-Blockers vs Non-Dihydropyridine Calcium Channel Blockers in Heart Failure With Preserved Ejection Fraction: A Systematic Review and Meta-Analysis

**DOI:** 10.7759/cureus.116345

**Published:** 2026-09-16

**Authors:** Salman A Othman, Mansuor Alanazi, Saad F Aljuadi, Bushra Moeed, Latifah K Albaiahy, Mahmoud M Emara, Bassem A Ahmed, Wafa N Alwabisi, Mehrab K Mohammed, Hanan S Alquthami, Alanoud T Alotaibi, Aya Abdelrahman, Manar A Asiri, Shaima A Almaadi, Ali S Metwaly

**Affiliations:** 1 Department of Pharmacology, Dr. Sulaiman Al Habib Medical Group, Riyadh, SAU; 2 Department of Family and Community Medicine, Faculty of Medicine, University of Tabuk, Tabuk, SAU; 3 College of Medicine, Prince Sattam bin Abdulaziz University, Al-Kharj, SAU; 4 Department of Pharmacology, Armed Forces Hospital Southern Region, Khamis Mushait, SAU; 5 Department of General Practice, King Saud bin Abdulaziz University for Health Sciences, Riyadh, SAU; 6 Department of Cardiology, National Heart Institute, Giza, EGY; 7 Department of Internal Medicine, Faculty of Medicine, Benha University, Banha, EGY; 8 Faculty of Medicine, University of Tabuk, Tabuk, SAU; 9 College of Pharmacy, Omdurman Islamic University, Omdurman, SDN; 10 Department of Critical Care Nursing, Ministry of National Guard Health Affairs, Taif, SAU; 11 College of Medicine, Imam Mohammad Ibn Saud Islamic University, Riyadh, SAU; 12 Faculty of Medicine, Cairo University, Giza, EGY; 13 College of Pharmacy, King Khalid University, Abha, SAU; 14 College of Clinical Pharmacy, King Faisal University, Hofuf, SAU; 15 Department of Precision Medicine, Faculty of Pharmacy, Alexandria University, Alexandria, EGY

**Keywords:** adrenergic beta-antagonists, calcium channel blockers, heart failure, meta-analysis, stroke volume

## Abstract

Determining the most effective medication regimen to manage blood pressure and heart rate in patients with heart failure with preserved ejection fraction (HFpEF) continues to lack a clear clinical consensus. Although beta-blockers (BBs) are widely prescribed, their negative chronotropic effects may worsen exercise intolerance. Alternative agents like verapamil and diltiazem, which belong to the non-dihydropyridine calcium channel blocker (non-DHP CCB) class, offer a physiological alternative, yet comparative data are scarce. A systematic review and meta-analysis were conducted, comparing BBs vs non-DHP CCBs in adults with HFpEF (left ventricular ejection fraction ≥ 50%). A literature search across key databases was conducted up until May 2026. Primary outcomes included all-cause mortality and heart failure (HF) hospitalization. Pooled hazard ratios (HRs) with 95% CIs were calculated using a random-effects analytical framework incorporating the Hartung-Knapp-Sidik-Jonkman variance correction. Evidence certainty was evaluated using the Grading of Recommendations Assessment, Development, and Evaluation (GRADE) framework. Of the 576 retrieved records, two observational cohorts (106,848 patients) met the inclusion criteria. Only one of the two eligible cohorts analyzed real patient-level data; the other was generated. The two-study pooled estimates are reported as exploratory: all-cause mortality HR 0.88 (95% CI 0.34-2.26; p = 0.336) and HF hospitalization HR 0.91 (95% CI 0.34-2.42; p = 0.446), with I² > 80% for both outcomes. At k = 2, these dispersion statistics are themselves unstable, and the pooled intervals are not clinically interpretable. Restricted to the single real-world cohort, the estimates were HR 0.83 (95% CI 0.80-0.85) and HR 0.98 (95% CI 0.91-1.05), respectively, which are the figures of one observational study and carry no meta-analytic weight. Cardiovascular mortality, major adverse cardiovascular events, exercise capacity, and quality of life were prespecified but reported by neither study in poolable form. The confidence in these findings was rated as very low because the data stem from observational research and exhibit significant statistical variability alongside wide CIs. Current evidence does not demonstrate the prognostic superiority of either BBs or non-DHP CCBs in HFpEF. Given the very low certainty of the available data and high clinical heterogeneity, pharmacological selection should remain individualized. High-quality randomized controlled trials are needed to inform clinical guidelines.

## Introduction and background

Heart failure with preserved ejection fraction (HFpEF) accounts for approximately half of all heart failure (HF) cases worldwide and is associated with substantial morbidity, frequent hospitalizations, and high mortality [1]. The pathophysiological complexity of HFpEF, characterized by varying degrees of diastolic dysfunction, left ventricular hypertrophy, impaired active relaxation, and arterial stiffness, makes its therapeutic management challenging [1,2]. HFpEF coexists with conditions such as hypertension and atrial fibrillation (AF), which are major causes of symptom exacerbation and disease progression [3,4]. Optimizing hemodynamics through effective heart rate and blood pressure control is the cornerstone of HFpEF management to improve ventricular filling and reduce myocardial oxygen demand [4].

Beta-blockers (BBs) have been the default pharmacotherapy for rate and blood pressure control in this population due to extrapolation from their undisputed survival benefits in patients diagnosed with HF with reduced ejection fraction (HFrEF) [4,5]. However, the pathophysiological differences between these two phenotypes raise questions regarding the ubiquitous use of BBs in HFpEF. A significant proportion of patients with HFpEF suffer from chronotropic incompetence; the negative chronotropic effects of BBs can impair exercise capacity by restricting the required augmentation of cardiac output during physical exertion [2,4], whereas utilizing non-dihydropyridine calcium channel blockers (non-DHP CCBs) such as verapamil and diltiazem offers an alternative that is mechanistically and physiologically sound. Non-DHP CCBs exert necessary rate-controlling properties while promoting peripheral vasodilation and improving left ventricular diastolic filling without the same degree of exercise-limiting chronotropic suppression [2,6].

Evidence from related clinical scenarios highlights the potential superiority, or at least noninferiority, of non-DHP CCBs over BBs. In the management of acute AF with a rapid ventricular response, a frequent precipitant of acute decompensation in HFpEF, several comparative studies have demonstrated that diltiazem achieves target rate control more rapidly and effectively than metoprolol, with comparable or superior safety profiles regarding hypotension and bradycardia [7-10]. Furthermore, in populations with decompensated HF and AF, diltiazem and metoprolol exhibit equal safety profiles without worsening the incidence of HF exacerbations [11]. In the outpatient setting, patients with chronic AF treated with non-DHP CCBs demonstrated maintained physical exercise capabilities and lower circulating levels of NT-proBNP when contrasted with patients receiving BB therapy, which impaired exercise tolerance [6].

In hypertension, foundational trials, such as the Nordic Diltiazem (NORDIL) study, established that diltiazem-based regimens are as effective as BB- or diuretic-based regimens in reducing cardiovascular morbidity and mortality [12]. Recent randomized crossover data suggest that substituting BBs with CCBs in patients with HFpEF yields better functional capacity and objective physical activity metrics [2]. Despite these mechanistic advantages, a recent meta-analysis of CCBs in HFpEF yielded inconclusive prognostic benefits; however, this was due to the pooling of DHP and non-DHP agents, which masked the unique AV-nodal blocking and lusitropic benefits of diltiazem and verapamil [1].

Despite clear mechanistic rationales and supportive data from acute and comorbid settings [3], current clinical guidelines provide ambiguous recommendations regarding the optimal choice of BBs vs non-DHP CCBs for patients with HFpEF [4,5]. To date, clinical decision-making has relied heavily on physician preference and extrapolated data rather than direct, high-quality comparative evidence. Therefore, this systematic review aimed to evaluate and, critically, characterize the availability and quality of comparative evidence for BBs vs non-DHP CCBs in HFpEF, rather than presume a sufficient evidence base for definitive comparison.

## Review

Methods

Protocol Registration and Reporting Standards

The methodology for this systematic review and its quantitative synthesis adhered to the guidelines outlined in the updated PRISMA 2020 statement [13]. The study protocol was prospectively registered with the International Prospective Register of Systematic Reviews (PROSPERO; CRD420261341761) to ensure methodological transparency and mitigate outcome reporting bias [14].

Search Strategy and Data Sources

We queried five primary online academic repositories (Scopus, Web of Science, Cochrane CENTRAL, Embase, and PubMed/MEDLINE) from database inception to May 2026. The search strategy combined controlled vocabulary (MeSH for PubMed/MEDLINE and Cochrane CENTRAL, Emtree for Embase) with free-text terms searched in title and abstract fields, joined using Boolean operators. Three concept blocks were combined with AND, with terms within each block combined with OR: (i) population, “heart failure with preserved ejection fraction”, “HFpEF”, “diastolic heart failure”, “diastolic dysfunction”; (ii) intervention, “beta-blocker”, “adrenergic beta-antagonists”, and named agents; and (iii) comparator, “calcium channel blockers”, “non-dihydropyridine”, “diltiazem”, “verapamil”. To minimize publication bias, the ClinicalTrials.gov trial registry was queried. No language or publication date restrictions were applied to the search.

Eligibility Criteria

Study selection was guided by the Population, Intervention, Comparator, Outcomes, Study design (PICOS) framework. The population included adult patients (≥18 years) with a confirmed diagnosis of HFpEF, defined by a left ventricular ejection fraction (LVEF) of 50% or greater, alongside verifiable clinical or echocardiographic indicators of elevated filling pressures or diastolic impairment. The intervention was treatment with any beta-adrenergic receptor antagonist (BB), and the prespecified comparator was treatment with a non-DHP CCB, specifically diltiazem or verapamil. During full-text assessment, one otherwise eligible study was identified in which the comparator was reported at the CCB class level and could not be disaggregated into DHP and non-DHP agents. The decision to retain that study, rather than exclude it, was taken post hoc and constitutes a deviation from the registered protocol; it was made because excluding it would have left a single eligible study. This deviation is reported here, with the resulting comparator indirectness carried into the Grading of Recommendations Assessment, Development, and Evaluation (GRADE) assessment and the limitations, rather than treated as satisfying the prespecified comparator criterion.

The primary outcomes were all-cause mortality, cardiovascular mortality, HF-related hospitalizations, and major adverse cardiovascular events (MACE), while the secondary outcomes included exercise capacity (peak oxygen consumption (VO₂) and six-minute walk test distance), health-related quality of life, and adverse events (e.g., symptomatic bradycardia and hypotension). Randomized controlled trials (RCTs) and observational cohort studies (utilizing confounder adjustment, such as techniques like inverse probability of treatment weighting or propensity score matching (PSM)) were eligible for inclusion. Unadjusted observational cohorts, case reports, and crossover trials without adequate washout periods were excluded.

Study Selection and Data Extraction

Article screening for titles and abstracts was performed by two distinct researchers independently using reference management software. Full-text articles of potentially eligible studies were reviewed against predefined inclusion criteria. Inter-rater reliability for study inclusion was quantified using Cohen’s kappa coefficient (κ) [15]; discrepancies were resolved through adjudication by a third investigator. Data extraction was performed independently using standardized, pre-piloted electronic forms to capture the study characteristics, baseline patient demographics, pharmacological interventions, and clinical outcomes.

Quality Assessment, Risk of Bias (RoB), and Certainty of Evidence

RoB was assessed independently by two reviewers at the study level. For observational studies, the Newcastle-Ottawa Scale (NOS) was used to assess patient selection, comparability of cohorts, and outcome ascertainment [16]. The overall certainty of the evidence for primary outcomes was evaluated using the GRADE framework, which downgrades evidence based on study limitations, inconsistency, indirectness, imprecision, and publication bias [17].

Statistical Analysis and Data Synthesis

All statistical analyses were performed using R software version 4.6.0 (R Foundation for Statistical Computing, Vienna, Austria) [18], utilizing the meta and metafor packages [19].

For time-to-event outcomes (e.g., mortality and HF hospitalization), pooled hazard ratios (HRs) with 95% CIs were calculated using the inverse-variance method. For dichotomous outcomes (e.g., incidence of adverse events), risk ratios or ORs were pooled. For continuous outcomes (e.g., exercise capacity), the mean difference or standardized mean difference was used, depending on the uniformity of the measurement scales.

To account for the anticipated clinical and methodological heterogeneity, all meta-analyses were performed using a random-effects (RE) model. Between-study variance (τ²) was estimated by restricted maximum likelihood (REML). REML maximizes the likelihood of the data after conditioning out the fixed effect, which removes the downward bias in τ² that arises when the pooled mean is estimated from the same data; it is approximately unbiased and is the default recommendation for continuous and log-transformed effect measures [20]. However, τ² is estimated from a single degree of freedom and is imprecise regardless of the estimator.

Conventional RE inference treats τ² as known once estimated and refers the pooled effect to a normal distribution, which understates uncertainty and inflates type I error when the number of studies is small. The Hartung-Knapp-Sidik-Jonkman (HKSJ) adjustment addresses this by rescaling the variance of the pooled estimate using the observed weighted dispersion of the study effects around it and by referring the result to a t-distribution with k − 1 degrees of freedom rather than the normal distribution. Statistical heterogeneity was quantified using the I² statistic and Cochran’s Q test, with I² values >50% indicating substantial heterogeneity [21]. Prediction intervals (PIs) were calculated to estimate the range in which the true effect of future studies is expected to fall.

Small-study effects and potential publication bias were assessed visually using contour-enhanced funnel plots and statistically evaluated using Egger’s regression test when 10 or more studies were included in a specific meta-analysis [22]. A two-tailed p-value <0.05 was considered statistically significant for all analyses.

Results

Literature Search and Study Selection

The systematic literature search yielded 576 records: 323 from PubMed/MEDLINE, 78 from Embase, 68 from Scopus, 48 from Web of Science, and none from Cochrane CENTRAL, together with 59 records from the ClinicalTrials.gov trial registry. After removal of 103 duplicate records, 473 citations underwent title and abstract screening. Of these, 411 records were excluded for not meeting the predefined inclusion criteria. Records were excluded at title and abstract screening for the following reasons: 113 did not concern an HFpEF or LVEF ≥50% population; 96 did not evaluate a BB intervention or a CCB comparator; 77 used an ineligible study design; 69 reported none of the prespecified outcomes; and 56 were reviews, editorials, or commentaries.

Full-text records were sought for the remaining 62 reports, of which 59 could not be obtained as assessable reports. These 59 records were entries retrieved from the ClinicalTrials.gov registry for which no linked publication, posted results, or downloadable protocol existed; they were classified as not retrieved rather than as registry records without results. Because registry entries without posted results contain no extractable outcome data, no eligible study reporting adjusted comparative effect estimates was lost at this stage. Three full-text reports were assessed for eligibility. One study was excluded because of an irrelevant comparator (use of DHP CCBs). Two observational cohort studies met all eligibility criteria and were included in the systematic review and quantitative meta-analysis. The complete study selection process is shown in the PRISMA 2020 flow diagram (Figure 1).

**Figure 1:**
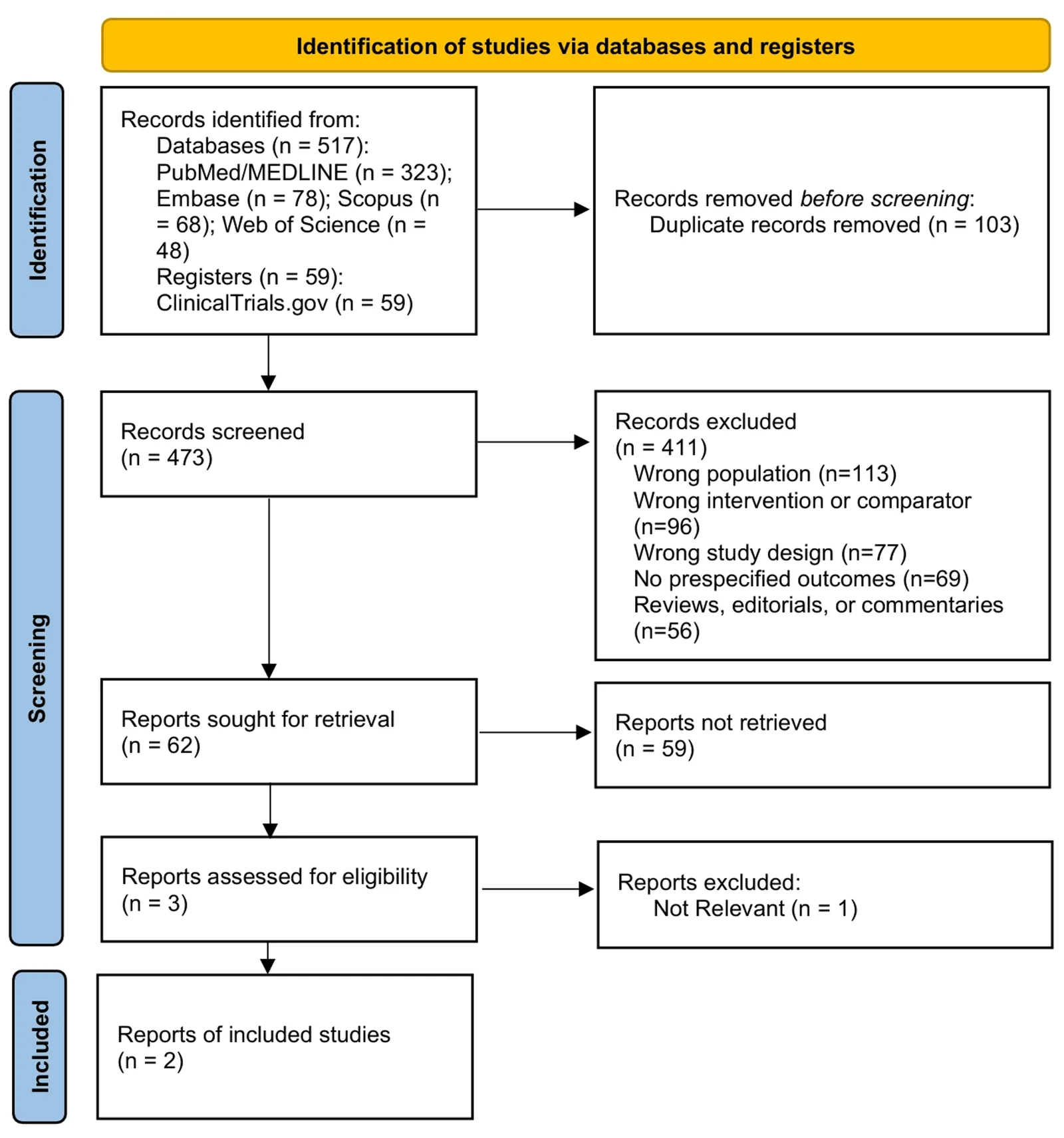
PRISMA 2020 flow diagram.

Study Characteristics

The baseline characteristics of the included studies are summarized in Table 1. Both studies were retrospective cohorts utilizing real-world data, contributing a combined total of 106,848 patients to the analysis [23,24]. Mansoor et al. [23] utilized a simulated electronic medical record (EMR)-derived cohort from Pakistan and Kyrgyzstan, employing 1:1 nearest-neighbor PSM to compare BBs with CCBs at a class level in 3,466 matched patients with HFpEF (LVEF ≥50%) with a mean follow-up of 5.2 years. Meyer et al. [24] analyzed a large United States Medicare fee-for-service claims database, comparing non-DHP CCBs (diltiazem/verapamil) with BBs in 102,382 beneficiaries aged ≥66 years with HFpEF and/or AF, with a mean follow-up of 1.9 years.

**Table 1:** Characteristics of eligible included studies. AF, atrial fibrillation; BB, beta-blocker; CCB, calcium channel blocker; Cox PH, Cox proportional hazards; CV, cardiovascular; DHP, dihydropyridine; EMR, electronic medical record; FFS, fee-for-service; HF, heart failure; HFpEF, heart failure with preserved ejection fraction; ITT, intention-to-treat; LVEF, left ventricular ejection fraction; non-DHP CCB, non-dihydropyridine calcium channel blocker; NYHA, New York Heart Association; PSM, propensity score matching

Study (country)	Design	Population/setting	Intervention vs comparator	Sample size (BB/CCB)	Outcomes extracted	Adjustment/follow-up
Mansoor et al. (2025) [23] (Pakistan/Kyrgyzstan)	Retrospective cohort (EMR-distribution-derived, propensity-score-matched)	Adults with HFpEF (LVEF ≥50%), NYHA I–IV; single military hospital EMR, 2014-2023	BB vs CCB (class-level)	2,000/2,000 (1,733/1,733 after PSM)	All-cause and CV mortality, HF hospitalization, composite endpoint, bradycardia, hypotension, discontinuation	1:1 nearest-neighbor PSM (caliper 0.2 SD), Cox PH; mean follow-up 5.2 years
Meyer et al. (2023) [24] (USA)	Retrospective new-user cohort (multivariable-adjusted, claims-based)	Medicare FFS beneficiaries ≥66 years with HFpEF and/or AF; 2014-2018	Non-DHP CCB (diltiazem/verapamil) vs BB	83,458/18,924	HF hospitalization, all-cause mortality (ITT and censored-at-switch)	Multivariable Cox (sociodemographics, comorbidities, frailty proxy, co-meds); mean follow-up 1.9 years

Two features of the included cohorts limit their correspondence to the prespecified review question. First, the Meyer et al. [24] cohort was assembled from claims-based diagnostic coding for HFpEF and/or AF, and the published report does not permit determination of the proportion of analyzed beneficiaries in whom HFpEF was confirmed against the echocardiographic or filling-pressure criteria required by our eligibility framework; an undetermined subset will therefore have had AF without confirmed HFpEF. Second, Mansoor et al. [23] compared BBs with CCBs at the class level, and the report does not specify the relative contribution of DHP vs non-DHP agents within the comparator arm. Both features constitute indirectness relative to the prespecified PICOS; both are carried forward into the GRADE assessment and the limitations, and neither can be quantified from the published reports. Both studies provided adjusted HRs for the outcomes of interest, which were harmonized during extraction to ensure that an HR <1 uniformly favored BBs.

One included study (Mansoor et al. [23]) did not analyze individual patient-level records but instead used a synthetically generated cohort (n = 4,000) produced in R from parametric distributions derived from single-center EMR data, with probabilistic treatment assignment (random seed 12345). It was retained on the grounds that it was the only identified report isolating a propensity-matched comparison of BBs vs CCBs restricted to an LVEF ≥50% population, and its exclusion would have reduced the review to a single study. We emphasize that this retention decision is a reporting choice, not a methodological claim: synthetically generated observations reproduce the distributional assumptions specified by their generating model and cannot confirm or refute a clinical hypothesis, so this cohort does not constitute primary clinical evidence, and its apparent precision is a function of the programmed sample size (n = 4,000) rather than empirical information. Accordingly, every pooled estimate reported below is exploratory and hypothesis-generating, and the contribution of this cohort is displayed separately through a leave-one-out procedure.

Risk of Bias and Quality Assessment

The RoB was evaluated using the NOS for cohort studies. Both studies achieved a total score of 7 out of 9 possible stars, indicating an overall good-to-fair methodological quality. Meyer et al. [24] received deductions in the comparability domain due to potential residual confounding inherent in claims-based analyses, whereas Mansoor et al. [23] lost points in the selection domains due to limitations surrounding EMR simulation representativeness and non-exposed cohort derivation. Detailed domain-level judgments are visualized in the NOS traffic-light plot (Figure 2) and risk-of-bias distribution summary (Figure 3).

**Figure 2:**
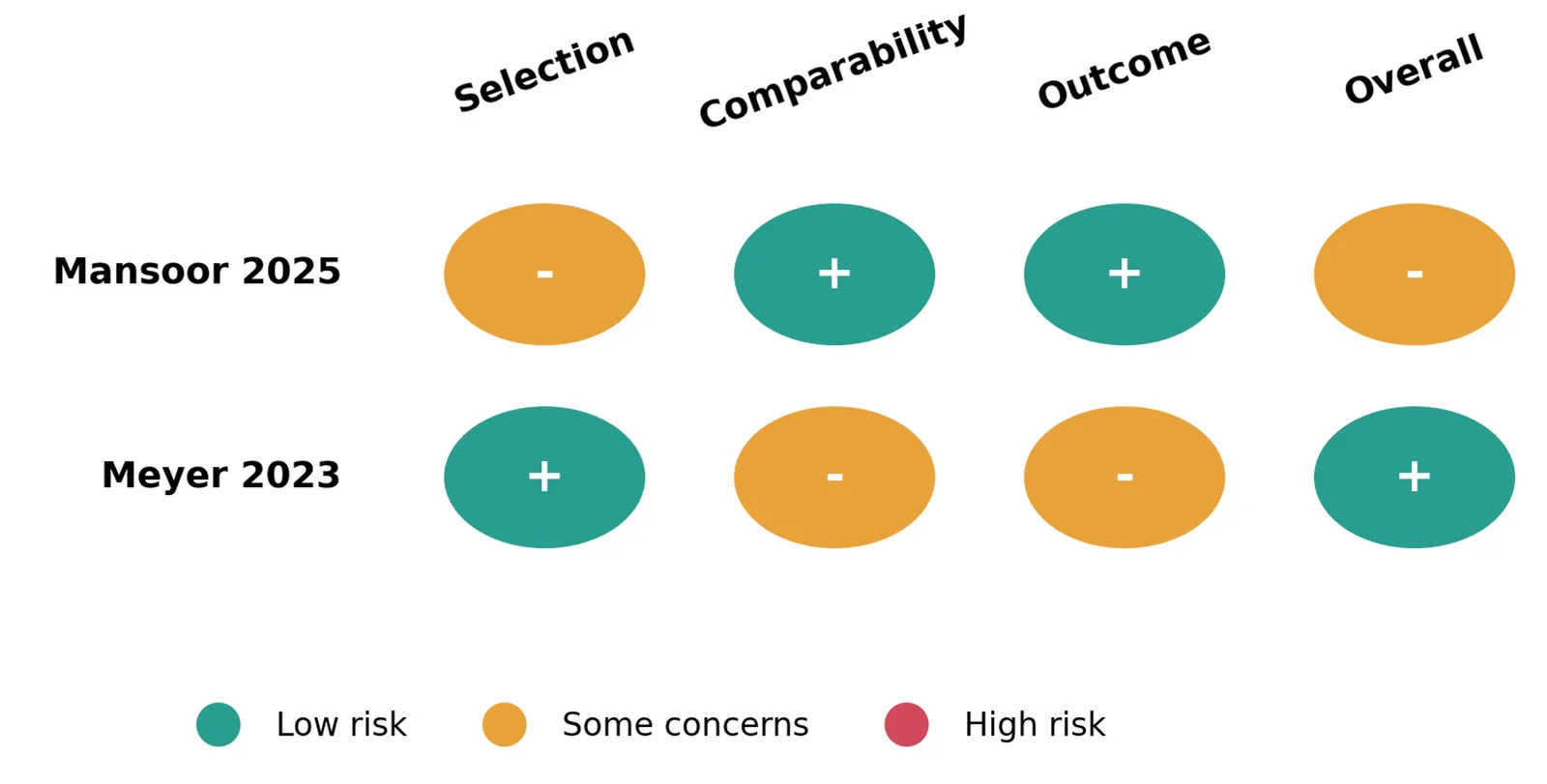
RoB (NOS) - traffic-light plot. This figure provides a study-level visual breakdown of bias judgments across Selection, Comparability, and Outcome domains [23,24]. NOS, Newcastle-Ottawa Scale; RoB, Risk of Bias

**Figure 3:**
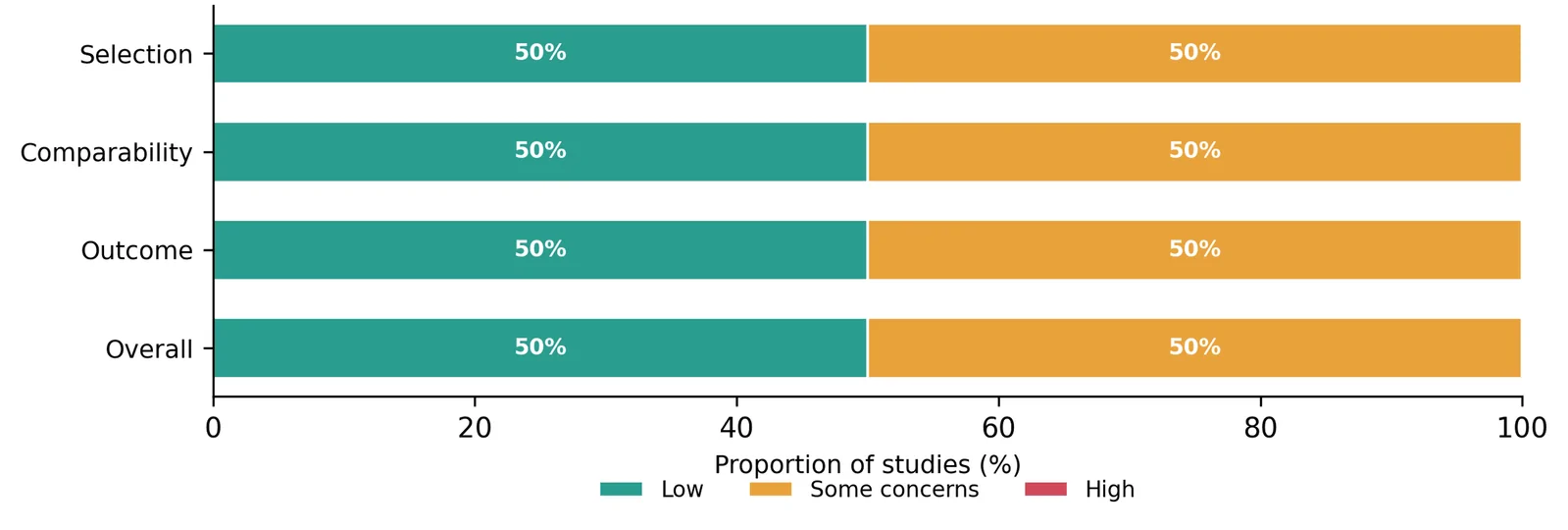
RoB judgment distribution by domain (NOS). This figure displays the overall proportional distribution of risk-of-bias judgments across the included cohort studies. NOS, Newcastle-Ottawa Scale; RoB, Risk of Bias

Primary Outcomes

Due to the limited number of studies (k = 2), RE models utilizing the REML estimator and HKSJ variance adjustment were employed. Of the prespecified outcomes, only all-cause mortality and HF hospitalization were reported by both eligible studies in a form permitting extraction of adjusted, directionally harmonized effect estimates. Neither study reported adjusted estimates for cardiovascular mortality, MACE, peak oxygen consumption, six-minute walk distance, or health-related quality of life in an LVEF ≥50% population; symptomatic bradycardia, hypotension, and treatment discontinuation were reported by Mansoor et al. [23] only and could not be pooled. These outcomes were not synthesized. The unavailability of comparative data for every prespecified secondary endpoint, and for the two prespecified cardiovascular composite endpoints, is reported here as a substantive finding rather than an omission.

All-Cause Mortality

Both studies reported adjusted HRs for all-cause mortality. The pooled RE quantitative synthesis showed that all-cause mortality did not differ significantly between patients treated with BBs and those treated with non-DHP CCBs (HR 0.88, 95% CI 0.34-2.26; p = 0.336). Substantial statistical heterogeneity was observed between the studies (I² = 82.2%, τ² = 0.0093, Cochran’s Q = 5.62, p = 0.0177) (Figure 4).

**Figure 4:**
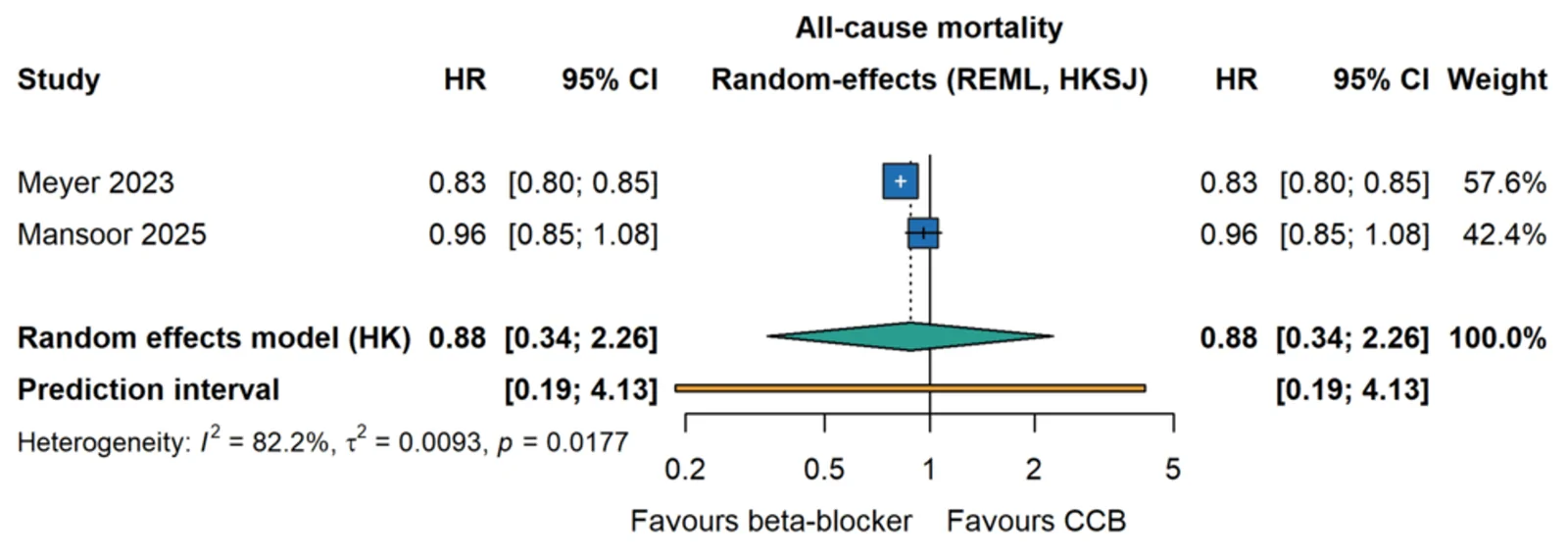
Forest plot - all-cause mortality. RE model (REML and HKSJ adjustment) comparing BBs vs non-DHP CCBs. HR <1 favors BBs [23,24]. BB, beta-blocker; CCB, calcium channel blocker; DHP, dihydropyridine; HKSJ, Hartung-Knapp-Sidik-Jonkman; HR, hazard ratio; RE, random-effects; REML, restricted maximum likelihood

HF Hospitalization

The risk of HF-related hospitalization did not differ significantly between the BB and non-DHP CCB treatment groups. The pooled HR was 0.91 (95% CI 0.34-2.42; p = 0.446). Substantial heterogeneity was also present for this outcome (I² = 80.1%, τ² = 0.0095, Cochran’s Q = 5.03, p = 0.0249) (Figure 5). A graphical summary of the pooled HRs across both primary outcomes is presented in Figure 6.

**Figure 5:**
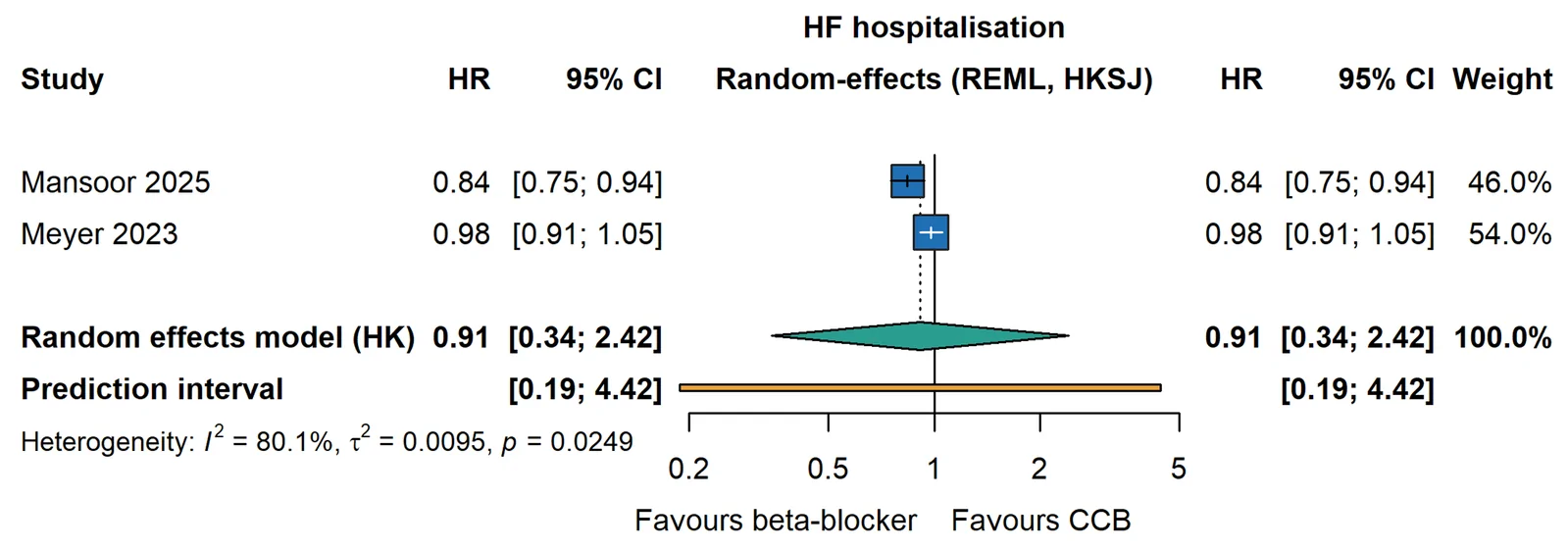
Forest plot - HF hospitalization. RE model (REML and HKSJ adjustment) comparing BBs vs non-DHP CCBs. HR <1 favors BBs [23,24]. BB, beta-blocker; CCB, calcium channel blocker; DHP, dihydropyridine; HKSJ, Hartung-Knapp-Sidik-Jonkman; HR, hazard ratio; RE, random-effects; REML, restricted maximum likelihood

**Figure 6:**
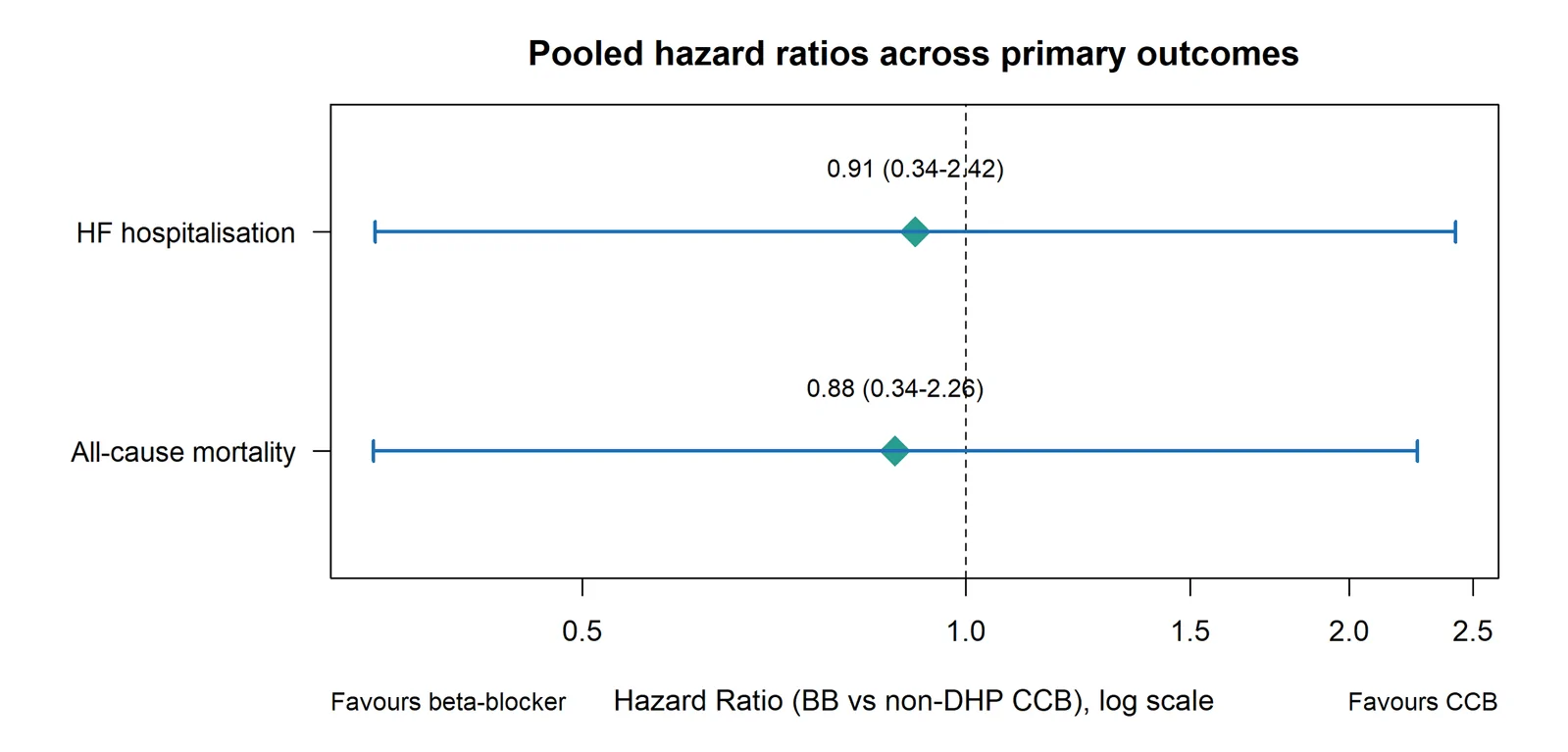
Pooled HRs across primary outcomes. Summary plot demonstrating the pooled RE HRs (log scale) for all-cause mortality and HF hospitalization. HF, heart failure; HR, hazard ratio; RE, random-effects

Because the synthesis was restricted to two studies, meta-regression, subgroup analysis, and formal small-study-effect testing were not possible. It must further be emphasized that at k = 2, the between-study variance is estimated from a single degree of freedom: τ², I², Cochran’s Q, and the PI are correspondingly unstable, and the values reported above describe the discordance between two individual estimates rather than reliably quantifying true between-study variability. For the same reason, the HKSJ-adjusted CIs are wide, and the pooled point estimates are not clinically interpretable as measures of comparative effect. A leave-one-out procedure was nonetheless performed to display the contribution of the simulated-data cohort. Removing Mansoor et al. [23] does not yield an alternative meta-analytic estimate; it reduces the evidence base to a single real-world claims cohort, that of Meyer et al. [24], whose full-cohort intention-to-treat estimates were HR 0.83 (95% CI 0.80-0.85) for all-cause mortality and HR 0.98 (95% CI 0.91-1.05) for HF hospitalization. These figures are the result of one observational study and are presented as the most clinically interpretable data available, not as evidence that heterogeneity was resolved or as a demonstration that BBs are superior. The dispersion observed in the two-study synthesis reflects no more than the fact that the two available estimates disagree, one of them having been generated from prespecified distributional assumptions rather than from observed patient outcomes.

Certainty of Evidence (GRADE)

The certainty of evidence for both primary outcomes (all-cause mortality and HF hospitalization) was evaluated using the GRADE framework and was determined to be very low (Table 2). The initial evidence certainty was low due to the observational nature of the included cohorts. It was downgraded by one level for RoB (simulated data in one cohort; residual confounding and immortal-time bias in the other), one level for inconsistency (substantial heterogeneity, I² >80%, with effect estimates in opposing directions), one level for indirectness (population indirectness in Meyer et al. [24], whose cohort was defined as HFpEF and/or AF, and comparator indirectness in Mansoor et al. [23], whose comparator was class-level CCB rather than non-DHP CCB), and one level for imprecision (very wide 95% CIs crossing the line of no effect). As certainty cannot fall below very low, the four downgrades leave the final rating at very low for both outcomes, but the number of domains downgraded should be read as an indication of how far the available evidence sits from the review question. Publication bias was not suspected to be a downgrading factor (not serious) but could not be formally assessed.

**Table 2:** GRADE summary of findings: BBs vs non-DHP CCB in HFpEF. Both outcomes derive from adjusted observational cohorts, so the baseline GRADE certainty starts at low. The final certainty reflects net downgrading for risk of bias, inconsistency, indirectness, and imprecision. ᵃ Downgraded one level for risk of bias: Both included studies were observational. Meyer et al. [24] utilized claims-based ascertainment susceptible to residual confounding; Mansoor et al. [23] utilized simulated data. ᵇ Downgraded one level for inconsistency: Unexplained and substantial statistical heterogeneity was observed between the two studies (I² >80% for both outcomes), with effect estimates trending in opposite directions. ᶜ Downgraded one level for indirectness: The population of Meyer et al. [24] was defined by claims coding for HFpEF and/or AF, with an indeterminable proportion having AF without confirmed HFpEF; and the comparator of Mansoor et al. [23] was CCBs at the class level rather than non-DHP agents specifically. ᵈ Downgraded one level for imprecision: The 95% CIs utilizing the HKSJ adjustment are exceedingly wide and cross the line of no effect (1.0), indicating high uncertainty in the true effect size. ᵉ Publication bias: Not formally assessable due to k <10 but not strongly suspected based on the retrieved records. AF, atrial fibrillation; BB, beta-blocker; CCB, calcium channel blocker; DHP, dihydropyridine; GRADE, Grading of Recommendations Assessment, Development, and Evaluation; HF, heart failure; HKSJ, Hartung-Knapp-Sidik-Jonkman; RoB, risk of bias

Outcome (BB vs non-DHP CCB)	No. studies (patients)	Pooled effect (95% CI)	RoB	Inconsistency	Indirectness	Imprecision	Publication bias	Certainty (GRADE)
All-cause mortality	2 (106,848)	HR 0.88 (0.34-2.26)	Serious ↓ᵃ	Serious ↓ᵇ	Serious ↓ᶜ	Serious ↓ᵈ	Not serious ᵉ	⊕◯◯◯ Very low
HF hospitalization	2 (106,848)	HR 0.91 (0.34-2.42)	Serious ↓ᵃ	Serious ↓ᵇ	Serious ↓ᶜ	Serious ↓ᵈ	Not serious ᵉ	⊕◯◯◯ Very low

Discussion

To our knowledge, the current study represents the initial systematic review and meta-analysis dedicated to directly evaluating the clinical endpoints of non-DHP CCBs vs BBs specifically among patients with HFpEF. Synthesizing data from over 106,000 patients across two large observational cohorts, our analysis demonstrated no statistically significant difference between the two pharmacological classes in terms of all-cause mortality or HF hospitalization. However, the pooled effect estimates were accompanied by exceptionally wide CIs and substantial statistical heterogeneity (I² >80%). The certainty of evidence for both outcomes was very low. Given that the synthesis rests on two studies with opposing effect directions, only one of which analyzed observed patient outcomes, the pooled estimates should be read as a characterization of the evidence gap rather than as an estimate of comparative effect; the true comparative effect of these agents remains undetermined.

The absence of definitive class superiority in our pooled analysis was driven by the discordant findings of the two included studies, which utilized different methodological approaches and data sources. Meyer et al. [24] analyzed a large Medicare fee-for-service claims database. In the full-cohort intention-to-treat estimates extracted and harmonized for this review, in which HR <1 favors BBs, non-DHP CCB use was associated with a higher risk of all-cause mortality (HR 0.83, 95% CI 0.80-0.85) and with a risk of HF hospitalization that did not differ materially between classes (HR 0.98, 95% CI 0.91-1.05). Mansoor et al. [23] used a propensity-score-matched cohort derived from simulated EMR distributions, in which BBs were associated with fewer HF hospitalizations (HR 0.84, 95% CI 0.75-0.94) and with mortality not significantly different from CCBs (HR 0.96, 95% CI 0.85-1.08). Therefore, the discordance between the two studies is confined to the mortality endpoint, where the estimates point in opposing directions relative to the null, and it is this discordance that drives the heterogeneity.

The statistical heterogeneity (I² = 82.2% for mortality; 80.1% for HF hospitalization) is a direct reflection of clinical and methodological disparities. Claims-based analyses, such as Meyer et al. [24], are susceptible to residual confounding and lack granularity regarding specific echocardiographic parameters (e.g., exact LVEF, E/e′ ratio) or natriuretic peptide levels. Furthermore, confounding by indication plays a major role in real-world prescribing patterns: patients prescribed BBs often have a higher burden of ischemic heart disease or established AF, whereas non-DHP CCBs might be reserved for patients with severe reactive airway disease or those intolerant to BBs. While Mansoor et al. [23] attempted to mitigate this via PSM, their reliance on EMR-simulated data restricts the generalizability and true real-world validity of their findings.

The choice of rate-controlling agents for HFpEF remains one of the most prominent dilemmas in contemporary cardiovascular medicine. HFpEF is characterized by a stiff, noncompliant left ventricle that depends on adequate diastolic filling time and an appropriate chronotropic response to exercise [1,25]. From a purely physiological standpoint, the negative chronotropic effect of BBs can unmask or exacerbate chronotropic incompetence, leading to severe exercise intolerance, a hallmark symptom of HFpEF [26]. Non-DHP CCBs, particularly diltiazem, exhibit a more favorable hemodynamic profile: they provide necessary rate control (especially in comorbid AF) while exerting peripheral vasodilatory and lusitropic effects that may enhance diastolic filling without blunting exercise-induced heart rate augmentation [2,27].

Despite these mechanistic theories, our pooled clinical data do not substantiate the routine replacement of BBs with non-DHP CCBs. Until definitive evidence emerges, clinical decision-making must remain individualized. For patients with HFpEF and recent myocardial infarction, active angina, or HFrEF-recovered phenotypes, beta-blockade remains indicated [4,5]. In patients with HFpEF exhibiting chronotropic incompetence, uncontrolled hypertension, or severe chronic obstructive pulmonary disease, a transition to diltiazem or verapamil may be a highly rational and safe alternative [3,24].

This study has several major limitations that must be acknowledged. First and foremost is the paucity of eligible studies; only two observational cohorts met the strict inclusion criteria for isolating an LVEF ≥50% population. Meta-analyses with k = 2 are highly sensitive to the individual weight of the included studies, precluding the use of meta-regression, subgroup analyses, or formal assessments of publication bias (e.g., Egger’s test). Also, both included studies were observational. Despite employing advanced statistical adjustments (multivariable Cox regression and PSM), residual confounding and immortal-time bias cannot be definitively ruled out. Second, the outcome coverage of the available evidence is extremely narrow. Cardiovascular mortality, MACE, exercise capacity, and health-related quality of life, the endpoints on which the mechanistic argument for non-DHP CCBs in HFpEF principally rests, were not reported in extractable adjusted form by either eligible study. Therefore, the comparison rests on two hard endpoints and cannot speak to the functional outcomes that motivate the clinical question.

A fundamental limitation is that one of only two eligible studies did not derive from observations on real patients: Mansoor et al. [23] analyzed a synthetically simulated cohort generated from aggregate EMR-derived distributions rather than individual patient records. Because synthetic data reproduce prespecified distributional assumptions rather than empirical outcomes, this cohort cannot be regarded as primary clinical evidence, and its narrow CIs reflect the programmed sample size (n = 4,000) rather than genuine precision. The pooled two-study estimates should be viewed as exploratory and hypothesis-generating only, and the leave-one-out analysis restricted to real-world data represents the more clinically interpretable result. A further limitation is that the two exposures being compared were not defined identically across the included studies. Meyer et al. [24] evaluated non-DHP agents specifically, whereas Mansoor et al. [23] compared BBs with CCBs as a class, without reporting the proportion of DHP agents in the comparator arm. To the extent that DHPs contributed to that arm, the pooled estimate does not isolate the AV-nodal blocking and lusitropic properties that provide the mechanistic rationale for this review, the precise limitation we identified in the existing CCB literature. Variations in dosage, specific agent pharmacokinetics (e.g., carvedilol vs metoprolol; diltiazem vs verapamil), and the presence or absence of concomitant AF likewise could not be adjusted for in the pooled analysis.

## Conclusions

The principal finding of this review is the near-absence of real-world comparative evidence. A systematic search identified only one study analyzing observed patient-level data; the second eligible cohort was synthetically generated and cannot serve as primary clinical evidence. Therefore, the pooled estimates reported here are exploratory, and this review should be read as a characterization of a critical evidence gap rather than as an evaluation of comparative efficacy. No prognostic superiority of either drug class is established, and none can be established from the literature. The lack of randomized data highlights a critical gap in the contemporary HF guidelines. Large-scale, head-to-head RCTs are required to determine the optimal pharmacological strategy for hemodynamic and rate control in the expanding and vulnerable HFpEF populations.
